# Wounds of the Scalp United by the Hair

**Published:** 1861-01

**Authors:** 


### Wounds of the Scalp United by the Hair.

In a letter to the editor of the American Medical Monthly, Dr. H. G. Davis, of New York, gives what he thinks an improvement upon the plan of Drs. Campbell and Pitts, for the union of wounds of the scalp by the hair instead of sutures. The improvement consists in attaching the hair in connection with the wound, on each side, to adhesive plaster, instead of tieing wisps of hair of the opposite sides of the wound, so as to keep the edges together.

Dr. Davis's plan is as follows:

"The hairs upon the edge of the wound, to the distance of one-sixteenth of an inch from the edge, are separated from the other hairs, combed straight, then cut off, leaving the portion attached to the scalp about an inch long. These are to be laid upon the adhesive surface of a strip of adhesive plaster about two inches long, of any width you choose, according to the direction you carry the plaster that is to be attached to it. After the hair is pressed down upon the adhesive surface, another strip is to be applied over it of the same width, and of sufficient length to pass over the head to the skin beyond the hair—as upon the forehead, temples, or back of the ear—when it can be secured to the skin.—This arrangement of the hair upon the adhesive plaster enables the surgeon to bring the edges of the wound in perfect coaptation without the slightest irritation. To illustrate the application, I will state a case:

"A gentleman was thrown over the side of his carriage into the road, striking the anterior and superior portion of his head upon a sharp stone; the scalp was divided to the length of four inches and a half—the cut being of a crescent shape—and separating the scalp from the side of the head to the depth of two and a half inches. The wound was filled with gravel and the loose hairs divided by the stone.

"The wound was carefully cleansed by squeezing warm water from a sponge upon its surface, and the hairs removed, as far as possible, without touching the parts. When every foreign substance was removed, the flap was brought to its place, and secured upon the opposite side of the head; a cold water compress was kept applied. At the end of twenty-four hours, the separated scalp was found to have united perfectly by the first intention—no suppuration taking place at any point. Let me add, that no mode effecting the coaptation of parts will secure union by the first intention where the exposed surfaces have been rubbed with a sponge, or otherwise irritated."

